# LL37 promotes angiogenesis: a potential therapeutic strategy for lower limb ischemic diseases

**DOI:** 10.3389/fphar.2025.1587351

**Published:** 2025-04-23

**Authors:** Yingying Yang, Gang Wu, Yini Wang, Qiancheng Mao, Dongxu Zhang, Jitao Wu

**Affiliations:** Yantai Yuhuangding Hospital, Yantai, China

**Keywords:** antimicrobial peptide, LL37, VEGFA-PI3K/AKT/mTOR pathway, angiogenesis, lower limb ischemia

## Abstract

**Purpose:**

To study the angiogenic capacity of antimicrobial peptide LL37 (cathelicidin antimicrobial peptide), explore its molecular mechanisms, and provide new ideas for treating lower limb ischemic diseases.

**Methods:**

LL37 was applied exogenously to human umbilical vein endothelial cells (HUVECs), and its effects on cell proliferation, migration, and angiogenesis were assessed using Cell Counting Kit-8 (CCK-8), plate cloning, scratch, and angiogenesis assays. A mouse lower limb ischemia model was established, with LL37 injected intramuscularly on days 0, 4, and 8. Blood flow recovery was evaluated by laser Doppler flowmetry. Immunofluorescence staining detected cluster of differentiation 31 (CD31) and cluster of differentiation 34 (CD34) expression, while Hematoxylin and Eosin (H&E) staining assessed muscle cell morphology. Quantitative real-time polymerase chain reaction (qRT-PCR) and Western blotting analyzed gene and protein expression changes in HUVECs.

**Results:**

LL37 enhanced the proliferative, migratory, and pro-angiogenic abilities of HUVECs. It significantly improved blood flow recovery in ischemic limbs, with higher CD31/CD34 expression and more intact muscle morphology. qRT-PCR analysis demonstrated elevated expression of angiogenesis-related genes in LL37-treated HUVECs. Western blotting revealed increased vascular endothelial growth factor A (VEGFA) expression and enhanced phosphorylation levels of the phosphatidylinositol 3-kinase (PI3K)/protein kinase B (AKT)/mammalian target of rapamycin (mTOR) pathway in LL37-treated cells.

**Conclusion:**

LL37 promotes angiogenesis via the VEGFA-PI3K/AKT/mTOR pathway, showing potential for treating lower limb ischemia by improving perfusion.

## 1 Introduction

Chronic limb ischemia (CLTI) is the end stage of peripheral arterial disease (PAD). It is a disease characterized by intermittent claudication, ulceration or gangrene of the limbs due to various reasons such as narrowing or occlusion of the arteries and inadequate blood perfusion (Farber, 2018; Beard, 2000). It is a problem with high morbidity and high healthcare costs worldwide. Of these, lower limb ischemic disease is the most common type (Levin et al., 2020). Currently, there is no standardized treatment for CLTI in most countries, and treatment varies considerably from region to region, resulting in CLTI still being associated with higher rates of amputation. Surgical bypass and endovascular therapy are the main blood supply reconstruction strategies for the treatment of CLTI (Cheng and Farber, 2024; Zeller et al., 2009). However, conservative treatments are more in line with the needs of some patients due to the limitations of operators, patients’ arterial disease patterns, surgical risks, available autologous venous bypass access, and patient preferences, placing new demands on the search for appropriate drugs (Desai et al., 2024).

Cathelicidin, a family of antimicrobial peptides (AMPs), is a critical component of the body’s innate immune system and is widely expressed across species. These small bioactive polypeptides are induced in response to infection and inflammation (Agier et al., 2015; Zhang Q. et al., 2023). In humans, cathelicidins are primarily represented by two major groups: defensins and the sole cathelicidin-derived peptide, LL37 (Fry, 2018; Izadpanah and Gallo, 2005). The name “LL37” reflects its structure—a 37-amino acid peptide originating from the N-terminal cleavage of the cathelicidin precursor protein, with leucine (L) as its first two residues.

LL37 exhibits broad-spectrum antimicrobial activity against Gram-positive and Gram-negative bacteria, fungi, and viruses (Luo et al., 2019; Rashki et al., 2022). I Beyond its direct microbicidal effects, LL37 plays a multifaceted role in immune regulation, including modulation of inflammatory mediators, immune cell chemotaxis, cytokine release, endotoxin neutralization, and even tumor suppression or promotion depending on the context (Chieosilapatham et al., 2018; Frasca and Lande, 2012).

The formation of new blood vessels is a prerequisite for tissue repair and improvement of ischemic symptoms (Veith et al., 2019). Angiogenesis is caused by various factors, from mechanical stress and hypoxia to the presence of soluble inflammatory mediators, and involves the germination of small capillaries (angiogenesis) and the growth of existing blood vessels (arteriogenesis) (Eelen et al., 2020; Kuwano et al., 2001). Emerging evidence highlights LL37 as a potent modulator of vascular growth. Studies have shown that LL37 promotes endothelial cell migration, tube formation, and VEGF-mediated signaling, accelerating wound healing and ischemic tissue recovery (Khung et al., 2015). For instance, LL37 activates formyl peptide receptor 2 (FPR2) and Epidermal Growth Factor Receptor/Extracellular Signal-Regulated Kinase (EGFR/ERK) pathways, mimicking pro-angiogenic factors like Vascular Endothelial Growth Factor (VEGF) (Tjabringa et al., 2003; Zhang H. et al., 2023). Additionally, LL37 synergizes with hypoxia-inducible factors (HIFs) to enhance perfusion in diabetic and ischemic models (Rodríguez-Martínez et al., 2008).

Despite these advances, the precise mechanisms by which LL37 influences functional angiogenesis and arterial growth remain incompletely understood. In this study, we investigate the role of LL37 in driving therapeutically relevant angiogenesis and evaluate its potential for treating ischemic diseases.

## 2 Materials and methods

### 2.1 Cell culture

Human umbilical vein endothelial cells (HUVECs) were purchased from the Shanghai Institute of Cell Biology, Chinese Academy of Sciences. HUVECs were cultured in DMEM medium supplemented with 10% FBS, 1% penicillin/streptomycin, and the cell lines were maintained at 37°C in a humidified 5% CO2 atmosphere. Cells were used between passages three and six to ensure consistent cell behavior, prior to treatment with: LL-37 (MCE, Cat# HY-P1222, ddH_2_O); VEGF165 (MCE, Cat#HEK293, 0.1% BSA); Control: phosphate-buffered saline (PBS) (Gibco, Cat# 10010001).

### 2.2 Cell Counting Kit-8 (CCK8) cell proliferation experiment

HUVECs were seeded in 96-well plates at 4,000 cells per well. LL37 was added to the experimental group to 5 μg/mL, and an equal volume of PBS was added to the control group. 10 μL CCK8 (Beyotime, Cat# C0038) was added to each well at 0, 12, 24, 36, and 48 h, and OD550 absorbance was measured 1 h later (Thermo Fisher Company, United States). Growth curves were plotted based on OD values to analyze the results. The experiment was repeated three times to ensure data reliability.

### 2.3 Clonogenic assay

HUVECs were seeded in 6-well plates at a density of 1,000 cells per well. LL37 was added to the experimental group to 5 μg/mL, and an equal volume of PBS was added to the control group. The medium was changed every 3 days, and cells were cultured for 12 days. 4% paraformaldehyde was added to each well to fix the cells for 20 min 700 μL of crystal violet (Beyotime, Beijing, China) was added to each well, left for 30 min at room temperature away from light. The number of clones >10 cells was counted by ImageJ. The experiment was repeated three times.

### 2.4 Angiogenesis test

Matrigel Basement Membrane Matrix (BD Biosciences) was melted overnight in a 4°C refrigerator. 50 μL/well of Matrigel gel was added to a pre-cooled 96-well plate and left at 37°C for 45 min to solidify. HUVECs were seeded at a density of 3 × 10^4 cells/well onto the Matrigel gel. The experimental group was incubated with complete medium containing LL37 (5 μg/mL) or VEGF165 (20 ng/mL), and the control group was incubated with complete medium only. Photographs were taken at 4, 6, and 10 h, and the number of tubes and branches were quantified using ImageJ software. The experiment was repeated three times.

### 2.5 Scratching experiment

HUVECs were seeded into six-well plates and cultured until the cell density reached approximately 90%. A ‘cross’ was drawn in the center of each well with a sterile 1 mL pipette tip. The wells were rinsed with PBS to remove cell debris, and fresh serum-free DMEM medium was added. LL37 (5 μg/mL) was added to the experimental group, while the control group received only fresh serum-free DMEM medium. Microscopic photographs were taken at 0, 24, and 48 h after the scratch, and the migration distance was measured. The experiment was repeated three times.

### 2.6 Transwell assay

Transwell assays were performed using Transwell inserts (8 µm pore size, BD Biosciences, United States) inserted into 24-well plates. For invasion assays, the upper chamber was coated with Matrigel (BD Biosciences, United States of America). The upper chamber was then coated with 200 µL of serum-free medium. In the experimental group, LL37 was added to a final concentration of 5 μg/mL, while no LL37 was added to the control group. The upper chamber contained either 2 × 10^4 cells (for migration assays) or 3 × 10^4 cells (for invasion assays). The lower chamber contained 700 µL of medium supplemented with 10% fetal bovine serum (FBS). After incubation at 37°C for 24 h, the cells were fixed with methanol, stained with 0.5% crystal violet, and observed and quantified under a light microscope.

### 2.7 Real-time quantitative RT‒PCR (qRT‒PCR)

Experimental HUVECs were treated with 5 μg/mL of LL37 for 24 h or 48 h, while control HUVECs were not treated with LL37. Total Ribonucleic Acid (RNA) was extracted from the HUVECs using a total RNA kit (Omega Biotek, Norcross, GA, United States of America) according to the manufacturer’s instructions. The RNA concentration was determined by measuring the ratio of absorbance at 260 nm to absorbance at 280 nm. The RNA was then reverse transcribed to cDNA using PrimeScript RT Master Mix (Zhongke Bioengineering Co., Ltd.). Quantitative PCR was performed using TB Green Premix Ex Taq II (Zhongke Bioengineering Co., Ltd.) on a Light Cycler 96 (Roche Group). The expression levels of the relevant genes were normalized to GAPDH, and the fold change was calculated using the comparative threshold cycling (ΔΔCt) method.

### 2.8 Animal culture

Clean-grade mice (C57BL/6) were purchased from Beijing Viton Lihua Laboratory Animal Technology Co. And housed under standard laboratory conditions (temperature 22°C ± 2°C, humidity 50%–60%, 12-h light/dark cycle). We used a non-invasive fluorescent labeling method to label and characterize the animal model. After shaving the dorsal fur of mice, numbering was performed using a 1% fluorescein sodium solution. Marking clarity was verified daily under UV light. The labeled area was maintained at least 1 cm away from the wound site (Klabukov et al., 2023). The study protocol was reviewed and approved by the Animal Ethics Committee of Yantai Yuhuangding Hospital (Approval Number 2025-YHD-036). This study report adheres to the ARRIVE 2.0 guidelines (Animal Research: Reporting of *In Vivo* Experiments) (Kilkenny et al., 2010).

### 2.9 Lower limb ischemia model in mice

Twenty-four 8-week-old C57/BL6J mice were randomly divided into three groups: Sham, Model, and LL37. After anesthesia and disinfection, a longitudinal incision was made from the groin to expose the femoral artery. In the Model and LL37 groups, the femoral artery was ligated, while the Sham group remained unligated. Quadriceps muscle injections were given on postoperative days 1, 4, and 8. The LL37 group received 25 μL of 5 μg/mL LL37 at 4 sites, and the Model group received an equal volume of PBS. Laser Doppler flow imaging was performed on days 0, 7, 14, and 21. The images allowed direct comparison of the pseudo-color images of the affected and the healthy side to quickly identify abnormal areas of perfusion. Quantitatively analyzed the perfusion situation by measuring the perfusion values of the injured and healthy limbs and calculating the ratio.

### 2.10 Hematoxylin-eosin (H&E) staining

Paraffin sections of mouse quadriceps muscle tissue were baked in an oven at 65°C for 45 min. Place in xylene I (15 min), xylene II (15 min), anhydrous ethanol I (10 min), anhydrous ethanol II (10 min), 95% ethanol (10 min), and 85% alcohol (10 min) for gradient dewaxing. Place in hematoxylin for 10 min and rinse with water. Place in 1% aqueous hydrochloric acid solution for a few seconds and rinse with water. Place in 1% ammonia solution for 1 min and rinse with running water. Place in eosin staining solution for a few seconds and rinse with running water. Place in 75% ethanol (2 min), 85% ethanol (2 min), anhydrous ethanol (5 min), anhydrous ethanol (5 min), xylene (5 min) for dehydration. The slices were sealed with neutral gum, dried naturally, and observed under the microscope.

### 2.11 Immunofluorescence

HUVECs were cultured in 6-well plates. The experimental group was treated with LL37 in complete culture medium for 48 h, while the control group was untreated. Cells were fixed with 4% paraformaldehyde for 20 min at room temperature. Cells were permeabilized with 0.5% Triton X-100 (diluted in PBS) for 20 min at room temperature. Cells were blocked with BSA for 2 h at room temperature. Sufficient primary antibodies were added: Mouse Anti-Human CD31 (Platelet Endothelial Cell Adhesion Molecule-1, PECAM-1) (Abcam, Cat# ab28364) and Rabbit Anti-Human CD34 (Hematopoietic Progenitor Cell Antigen CD34) monoclonal antibody (Abcam, Cat# ab81289). Incubated overnight at 4°C in a humidified chamber. After washing with PBS buffer 3 times, sufficient secondary antibodies were added: HRP-Sheep Anti-Rabbit Secondary Antibody (Abcam, Cat# ab7090) and HRP-Sheep Anti-Mouse Secondary Antibody (Abcam, Cat# ab47827). Incubated for 1 h at 37°C in the dark. After washing with PBS buffer 3 times, DAPI (Affinity Biosciences, United States) was added and incubated for 10 min in the dark. After washing with PBS buffer 3 times, the cells were observed and analyzed under a fluorescence inverted microscope.

### 2.12 Western blot assay

Cells were collected after trypsin digestion and centrifugation. Lysate was prepared by mixing RIPA lysate, protease inhibitor PMSF and phosphatase inhibitor NaF (NCM Biotech). Ensure that the final concentration of PMSF is 1 mM and the final concentration of NAF is 10 mM. collect the cells and add the lysate, 20–30 min on ice. After centrifuging and sonicating the supernatant, use the BCA technique (Beijing Tiangen Biotechnology Co., Ltd.) to determine the protein content. The protein was transferred to a PVDF membrane (Millipore, Bedford, MA, United States) after SDS-PAGE separation. Use β-actin and β-tublin as the loading control. After leaving it overnight in the primary antibody, let it sit in the secondary antibody for 2 h. Primary antibodies: VEGF-A (Rabbit monoclonal, Proteintech, Cat# 19003-1-AP, 1:6,000); PI3K (Rabbit polyclonal, Proteintech, Cat# 21739-1-AP, 1:5,000); Phospho-PI3K (Tyr458) (Rabbit monoclonal, Abmart, Cat# P42338, 1:1,000); AKT (Rabbit monoclonal, Cell Signaling Technology, Cat# 9272S, 1:1,000); Phospho-AKT (Ser473) (Rabbit polyclonal, Abcam, Cat# ab38449, 1:1,000); mTOR (Rabbit monoclonal, Cell Signaling Technology, Cat# 2972, 1:1,000); Phospho-mTOR (Ser2448)** (Rabbit monoclonal, Cell Signaling Technology, Cat# 2971, 1:1,000); β-Actin (Mouse monoclonal, Proteintech, Cat# 66009-1-Ig, 1:10,000); β-tubulin (Rabbit monoclonal, Cell Signaling Technology, Cat# 2146, 1:1,000); Secondary antibodies: Goat Anti-Mouse IgG-HRP (Abmart, Cat# M21001, 1:5,000); Goat Anti-Rabbit IgG-HRP (Abmart, Cat# M21002, 1:5,000). Using Image Quant LAS4000 (General Electric, Boston, Massachusetts, United States of America), protein bands were found, and ImageJ software was used to quantify them.

### 2.13 Statistical analysis

GraphPad Prism 8 software was used to analyze and graph the statistical results, and ImageJ software was used to count and calculate the relevant experimental results. All data were expressed as mean ± standard deviation (x ± s). Independent samples t-test was used for comparison of two independent groups (data verified for normality and chi-square); one-way/two-factor ANOVA supplemented by Tukey or Sidak *post hoc* tests was used for comparison of three and more groups. p < 0.05 was considered statistically significant. *P < 0.05, **P < 0.01, ***P < 0.001.

## 3 Results

### 3.1 Exogenous application of LL37 to HUVEC

**FIGURE 1:**
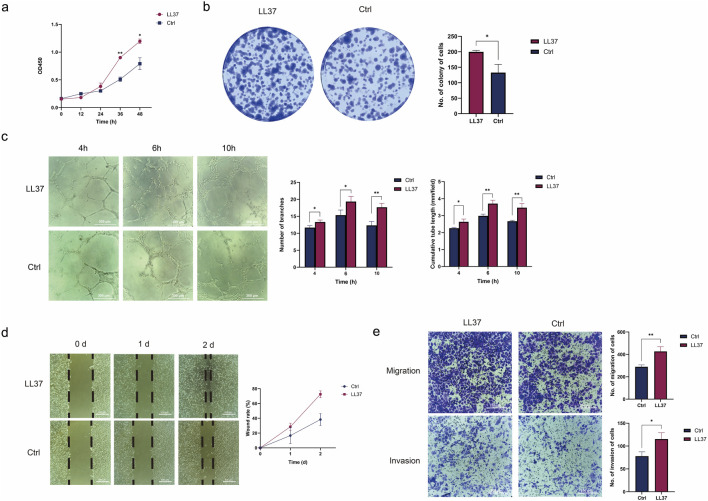
**(a)** The CCK8 assay examined the growth curves of control cells and LL37-treated HUVECs. **(b)** The plate cloning assay examined the proliferation of control cells and LL37-treated HUVECs. **(c)** The angiogenesis assay examined the angiogenesis of control cells and LL37-treated HUVECs. **(d)** The migration ability of control cells and LL37-treated HUVECs was detected by wound healing assay. **(e)** The capacity for migration and invasion of control cells and LL37-treated HUVECs was detected by transwell assay. Note: All experiments were independently repeated at least three times. *P < 0.05, **P < 0.01, ***P < 0.001, and ****P < 0.0001 indicate statistical significance.

HUVEC is sensitive to a variety of growth factors and can be used to assess the promotion of angiogenesis by LL37. The proliferative capacity of the cells was detected by the CCK-8 assay and plate cloning assay. The results showed that the OD value (450 nm) of HUVEC treated with LL37 for 48 h was significantly higher than Control group (P < 0.05) **(** Figure 1a). The number of plate clones of HUVEC treated with LL37 was significantly higher than Control group (P < 0.05) (Figure 1b).

After LL37 (5 μg/mL) was added to HUVECs, the angiogenesis results showed that both control and LL37 groups formed tubular structures after 4 h. The number of lumens formed in the LL37 group was higher, and statistical differences were found between the two groups by counting the number of branches and cumulative tube length (P < 0.05). After 6 h, the number of branches and cumulative tube length were significantly higher in the LL37 group compared with the control group (P < 0.01). After 10 h, part of the luminal structures in the control group disappeared, and the wall was defective and ruptured, while the luminal structures in the LL37 group remained intact, similar to the condition at 6 h. The number of branches (P < 0.01) and cumulative tube length (P < 0.05) were statistically significant between the two groups (Figure 1c), which indicated that LL37 could promote the angiogenic ability of HUVECs.

The results of the scratch assay showed that the average distance of cell migration was significantly greater in the HUVECs group with the addition of LL37 at both 24 h and 48 h (P < 0.01) (Figure 1d), suggesting that LL37 promotes the migratory ability of HUVECs. The results of the Transwell migration assay showed that the number of membrane-penetrating cells in the LL37-treated group was significantly higher than that in the control group (P < 0.05). This indicated that LL37 could enhance the migration ability of HUVEC. The Transwell invasion assay better simulated the environment that cells encounter the basement membrane and extracellular matrix during the invasion process *in vivo*. The results showed that the number of invaded cells in the LL37-treated group was significantly increased compared with that in the control group (P < 0.05), indicating that LL37 could promote the invasion ability of HUVEC (Figure 1e).

### 3.2 Endogenous application of LL37 to the mouse ischemia model

**FIGURE 2:**
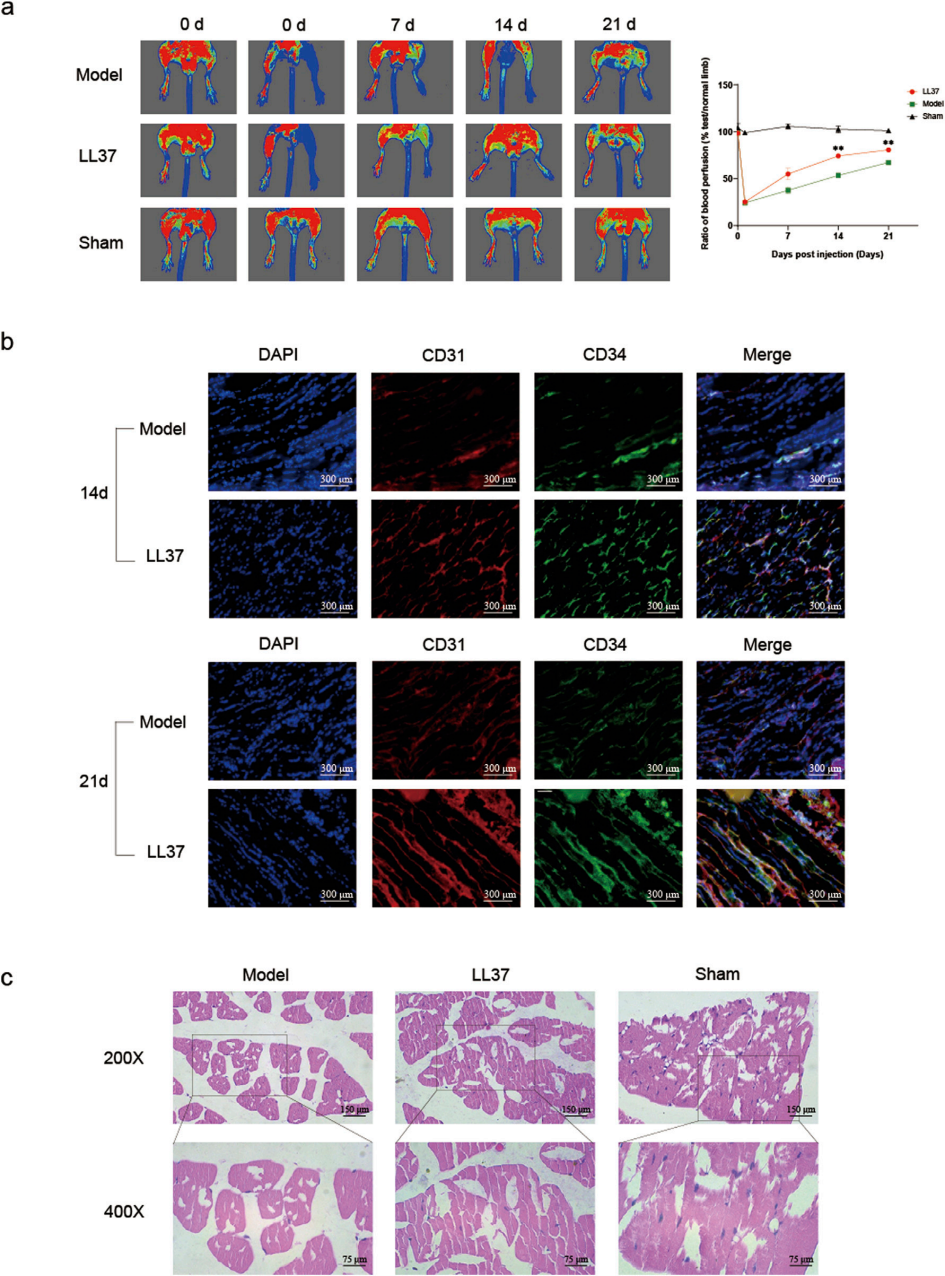
**(a)** Laser Doppler detection of lower limb blood perfusion in mice. Warmer colors (red or yellow) indicate higher perfusion, while cooler colors (blue) indicate lower perfusion. The perfusion values of the affected and healthy limbs were measured, and the ratio of the two was calculated for quantitative analysis. **(b)** Immunofluorescence detection of the expression of CD31 and CD34 in the lower limb muscles of mice. Blue fluorescence indicates nuclei, red fluorescence represents CD31, and green fluorescence represents CD34 (×200 magnification). **(c)** Observation of the morphology of mouse lower limb muscle cells using H&E staining. Note: *P < 0.05, **P < 0.01, ***P < 0.001, and ****P < 0.0001 indicate statistical significance.

The laser speckle blood flow imaging results showed that the blood flow in the lower limbs of the Sham surgery group mice remained normal. The blood flow in the lower limbs of the model group and LL37 group mice was normal before surgery, with no signs of hypoperfusion. On the second day after surgery, the blood flow perfusion in the lower limbs of mice in the model group and LL37 group decreased significantly compared to before surgery, and the blood flow in the affected limb accounted for only about 20% of the blood flow in the healthy side, indicating successful experimental modeling. On the seventh day after surgery, the LL37 group mice showed a recovery in lower limb blood flow perfusion, while the model group mice remained in a low blood flow perfusion state, but the difference between the two groups was not statistically significant (P > 0.05). On the 14th day after surgery, compared with the model group, the blood flow perfusion of ischemic limbs in the LL37 group mice was significantly improved, while the ischemic limbs in the model group mice were still in a state of low blood flow perfusion (P < 0.05). On the 21st day after surgery, the difference between the LL37 group mice and the model group was more significant (P < 0.05) (Figure 2a).

On the 14th and 21st postoperative days, frozen sections of the quadriceps muscle were prepared respectively using mice from the model group and the LL37 group. Immunofluorescence experiments showed that the expression of CD31 and CD34 in the muscle cells of the LL37 group was significantly stronger than that of the model group (Figure 2b).

We performed H&E staining on the quadriceps muscle of the ischemic lower limbs of mice in each group on the 21st postoperative day. The results showed that the muscle cell morphology in the quadriceps muscle of the Sham operation group was plump, and the cell outline was clear, with nearly no atrophic or apoptotic cells observed. The quadriceps muscle in the model group showed extensive atrophy and apoptosis. The diameter and size of muscle fibers were unevenly distributed, and most of the muscle fibers were morphologically atrophied with incomplete cell outlines. In contrast, only part of the muscle cells of the quadriceps in the LL37 group showed atrophy and apoptosis. The muscle morphology was more complete (Figure 2c). The experimental results indicated that LL37 could reduce the adverse effects of ischemia on muscle cells, maintain the basic morphology of the cells, and protect the muscle cells. The protective effect of LL37 on the cells is likely to be relevant for its improvement of blood perfusion, ensuring that muscle cells receive sufficient oxygen and nutrients to maintain normal metabolism and function.

### 3.3 LL37 might be involved in the VEGFA-pi3K/AKT/mTOR-signaling pathway

**FIGURE 3:**
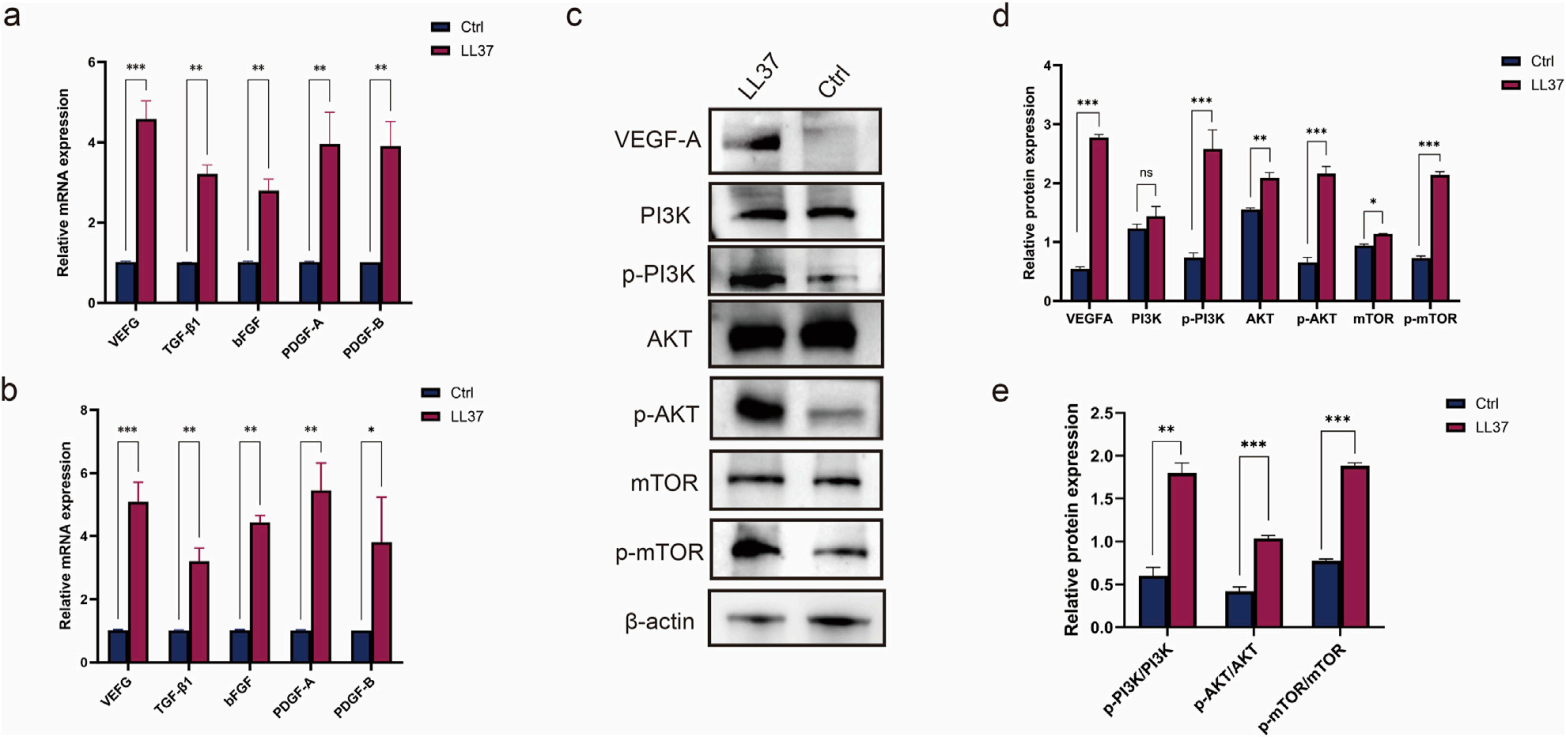
**(a)** qRT-PCR was used to detect the expression of angiogenesis-related genes in HUVECs treated with LL37 for 24 h **(b)** qRT-PCR was used to detect the expression of angiogenesis-related genes in HUVECs treated with LL37 for 48 h. **(c)** The changes in protein levels in HUVECs after treatment with LL37 were detected by Western blot. **(d)** Quantitative analysis of changes in protein expression levels in HUVECs after LL37 treatment. **(e)** Quantitative analysis of changes in protein phosphorylation levels in HUVECs after LL37 treatment.

After adding LL37 (5 μg/mL) into HUVECs culture medium, the expression levels of angiogenesis related genes in HUVECs were detected by qRT-PCR at 24 and 48 h, respectively. The results showed that after 24 h of LL37 treatment, the expression levels of Vascular Endothelial Growth Factor (VEGF), TGF- β (Transforming Growth Factor-β), bFGF (Basic Fibroblast Growth Factor), PDGF-A (Platelet-Derived Growth Factor Alpha Polypeptide and Platelet-Derived Growth Factor Subunit B (PDGF-B) were higher than those of the control group (P < 0.01); After 48 h, the above genes in the LL37 group were significantly elevated compared to the control group (P < 0.01) (Figures 3a,b), which proves that LL37 can promote the expression of angiogenesis related genes in HUVECs. The role of VEGFA on specific pathways was further explored by Western blotting (WB) (Supplementary Data Sheet 1). WB results showed that the application of LL37 was able to activate the PI3K/AKT/mTOR pathway in cells and significantly increase its phosphorylation level (Figure 3c). The expression of each relevant molecule of this pathway (Figure 3d) and its level of phosphorylation (Figure 3e) were quantitatively analyzed using ImageJ.

### 3.4 LL-37 versus VEGF165 in angiogenesis

**FIGURE 4:**
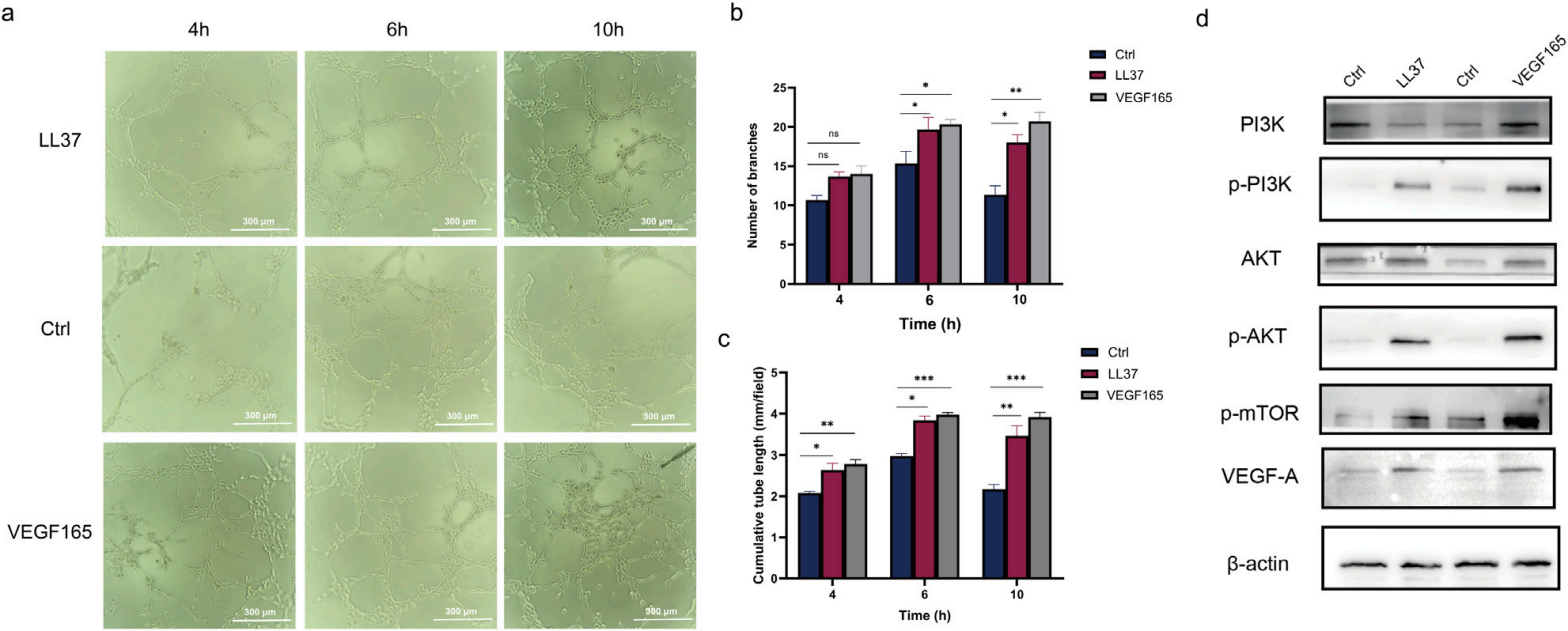
**(a–c)** The angiogenesis assay examined the angiogenesis of control cells and LL37-treated HUVECs. **(d)** The changes in protein levels in HUVECs after treatment with LL37 and VEGF165 were detected by Western blot.

By counting the number of branches and cumulative tube length, we found that both LL-37 and VEGF165 significantly enhanced endothelial tube formation compared to controls (p < 0.05, Figures 4a–c). Western blot analysis showed that LL-37 and VEGF165 induced comparable levels of phosphorylation of the VEGFA-PI3K/AKT/mTOR pathway after 1 h of treatment (Figure 4d; Supplementary Data Sheet 2).

## 4 Discussion

LL37 is a group of 37 amino acids at the N-terminus of the Cathelicidin protein, named after its initial amino acid L-L. As the only antimicrobial peptide present in the human body within the Cathelicidin family, it has been receiving attention for its bactericidal effect. LL-37 may disrupt the arrangement of membrane lipid molecules by interacting with amphiphilic phospholipid molecules at the N-terminus of the polymer, affecting the structure and function of the cell membrane, forming a so-called “carpet like” model that plays a bactericidal role. In addition to having direct antibacterial effects, it has also been found to have functions such as chemotactic immune cells, regulating the secretion of inflammatory promoting/inhibiting factors, coordinating natural and acquired immunity, and exerting immune regulatory effects. Currently, its clinical application is mainly limited to being a new type of antibacterial drug (Rashki et al., 2022; Boge et al., 2017; Geitani et al., 2022; Nagaoka et al., 2020).

Rebert Koczulla et al. first proposed the angiogenic effects of the human peptide antibiotic LL-37/hCAP-18 in 2003, stating that LL-37 may act on vascular endothelial cells and epithelial cells to stimulate angiogenesis (Koczulla et al., 2003). The key to the treatment of lower limb ischemic diseases is to promote angiogenesis to restore blood perfusion to ischemic tissues. Our study focused on the effects of LL37 on vascular endothelial cells to further expand its application in lower limb ischemic diseases (Lukasiewicz, 2016).

Proliferation and migration of vascular endothelial cells is the first step in vascular reconstruction and repair (Yan et al., 2011). CCK8 and plate cloning assay confirmed that LL37 could promote cell proliferation. The scratch assay showed that LL37 could promote the migration of endothelial cells. The HUVEC matrix gel angiogenesis assay more visually demonstrated the promotional effect of LL37 on endothelial cell angiogenesis. Quantify the number of branches and cumulative tube length. Branch number reflects the ability of cells to form complex networks and usually correlates with their ability to migrate and adhere. Cumulative tube length reflects the overall ability of cells to form tubular structures and is an important indicator for assessing angiogenic activity. After treatment with LL37, HUVEC exhibited a higher branching number and longer cumulative tube length, indicating that the cells possessed an enhanced ability to migrate and form tubular structures (Figure 1).

High ligation of the femoral artery with dissection was used to establish a stable lower limb ischemia model. This method was less traumatic to mice, and the ischemic state was obvious, stable, and long-lasting. Laser Doppler flowmetry was performed before and after the operation. The results showed that the mice in the model and experimental groups exhibited a significant decrease in the perfusion of the right lower limb, and a decrease in the intensity of astigmatism without disappearance, indicating that the modeling was successful. This approach was used to simulate the lower limb ischemic disease state. Laser Doppler was used to visualize the lower limb perfusion before, 1 week, 2 weeks and 3 weeks after ischemia, and the comparative results showed that LL37 treatment significantly improved the lower limb perfusion in mice (Figure 2).

CD31 is an adhesion molecule expressed on the surface of a variety of cells including vascular endothelial cells, platelets, and monocytes. It plays an important role in maintaining the normal function of vascular endothelial cells and in angiogenesis (DeLisser et al., 1997; Figueiredo et al., 2019). CD31 is currently the single best marker of endothelial differentiation and is expressed in 90% of hemangiosarcomas, hemangioendotheliomas, hemangiomas, and Kaposi’s sarcomas (Handra-Luca, 2019; Venkataramani et al., 2018). CD34 is a highly glycosylated transmembrane glycoprotein expressed mainly on the surface of hematopoietic stem cells and vascular endothelial cells. It plays a key role in vascular development and hematopoiesis and is involved in cell adhesion and signaling (Díaz-Flores et al., 2022). CD34 has a higher sensitivity to smaller, immature micro vessels and therefore may be more accurate in assessing micro vessel density (Mathiou et al., 2023). We respectively assessed the expression of CD31, CD34 at 14 and 21 days postoperatively and found that the expression of both molecules was significantly increased after LL37 treatment compared to the model group. It suggested that LL37 had a potential role in promoting angiogenesis, enhancing endothelial cell function and reducing endothelial function impairment (Figure 2).

The morphology of muscle cells is one of the most visual results of blood perfusion. Obstruction of blood supply to the muscle leads to clinical manifestations such as ischemia, hypoxia, nutritional deficiencies, and metabolic disorders, which in turn cause muscle atrophy, pain, and limitation of movement. If left untreated, it may lead to localized muscle necrosis or even paralysis (Fales et al., 1962). H&E staining was used to observe the morphology of muscle cells in the lower limbs. After adding LL37 treatment, the diameter and size distribution of muscle fibers were more uniform than control group, and atrophy and necrosis were not obvious. It indicated that the addition of LL37 could promote the recovery of muscle morphology and function. This change may be due to the promoting effect of LL37 on angiogenesis, which further increased the blood perfusion of the muscle tissue and improved the nutrient supply and oxygenation status of the muscle (Figure 2).

Angiogenesis is a complex process involving the interaction of multiple molecules and signaling pathways. VEGF is one of the most important regulators of angiogenesis, and the VEGF-A/VEGFR-2 signaling pathway is the most critical signaling pathway in physiological and pathological angiogenesis, which stimulates endothelial cell mitosis, chemotaxis, and morphogenesis, and induces tissue angiogenesis (Melincovici et al., 2018; Wang et al., 2021). TGFB1, bFGF, and PDGF are also important factors in angiogenesis, and are able to stimulate endothelial cell proliferation, migration, and vascular lumen formation, thus participating in the process of angiogenesis and remodeling (He et al., 2024; Lei et al., 2024; Ren et al., 2019; Xie et al., 2014). PCR results showed that HUVEC was treated with LL37, and the expression of VEGF, TGF-β1, bFGF, PDGF-A and PDGF-B were significantly elevated (Figure 3).

VEGF binds through its receptor VEGFR-2, which is an important upstream of the PI3K/AKT/mTOR pathway (Adamičková et al., 2023; Jia et al., 2023; Manjunathan et al., 2021). HUVEC treated with LL37 showed a significant increase in the expression level of VEGF, and furthermore, the phosphorylation level of the PI3K/AKT/mTOR pathway was found to be significantly elevated in the cells by WB (Figure 3). PI3K/AKT/mTOR pathway plays an important role in cell proliferation, survival, metabolism and angiogenesis. Activation of this pathway promotes endothelial cell proliferation and migration, enhances vascular permeability, and participates in angiogenesis (Ersahin et al., 2015; Karar and Maity, 2011).

Activation of PI3K produces phosphatidylinositol-3,4,5-trisphosphate (PIP3), which in turn activates AKT. activation of AKT promotes cell survival and proliferation, and inhibits apoptosis (Cheng et al., 2022). mTOR is a downstream target of AKT, and is divided into two complexes, mTORC1 and mTORC2. mTORC1 promotes protein synthesis and cell proliferation by phosphorylating S6K1 and 4 E-BP1. mTORC1 is also an active pathway in endothelial cell proliferation, and is involved in angiogenesis. And cell proliferation (Napolitano et al., 2022; Szwed et al., 2021). mTORC2, on the other hand, further enhances AKT activity by phosphorylating the S473 site of AKT (Fu and Hall, 2020; Guri et al., 2017). LL-37 recapitulated the proangiogenic effects and pathway activation characteristics of VEGF165 (Figure 4), suggesting that LL-37 may function as a VEGF mimetic peptide. However, further studies are needed to determine whether LL-37 acts through direct binding to VEGFR2 or through alternative receptors. Notably, the antimicrobial properties of LL-37 may further position it as a dual-function therapeutic candidate for infected wounds requiring concurrent angiogenesis and infection control. Future studies should explore whether LL-37 directly interacts with VEGF receptors or synergizes with endogenous VEGF.

It is noteworthy that LL-37 may indirectly promote angiogenesis through macrophage recruitment/activation (Zhang W. et al., 2023). However, our study has unequivocally demonstrated its direct mechanism of action using purified endothelial models and targeted pathway inhibition experiments. The potential synergistic effects between these direct and indirect mechanisms require further investigation.

## 5 Conclusion

LL37 can exert its pro-angiogenic effect by increasing the expression of VEGF in cells and enhancing the phosphorylation level of PI3K/AKT/mTOR pathway. We hope that it will play a therapeutic role in lower limb ischemic diseases.

## Data Availability

The original contributions presented in the study are included in the article/Supplementary Material, further inquiries can be directed to the corresponding author.

## Supplementary material

SUPPLEMENTARY DATA SHEET 1 The changes in protein levels in HUVECs after treatment with LL37 were detected by Western blot.

SUPPLEMENTARY DATA SHEET 2 The changes in protein levels in HUVECs after treatment with LL37 and VEGF165 were detected by Western blot.
