# Supplementary figures and images for: LL37 promotes angiogenesis: a potential therapeutic strategy for lower limb ischemic diseases
Source: Front Pharmacol. 2025 Apr 23;16:1587351. doi: 10.3389/fphar.2025.1587351 (PMC12055537; doi:10.3389/fphar.2025.1587351)

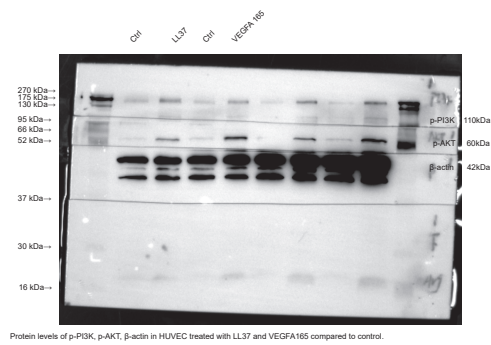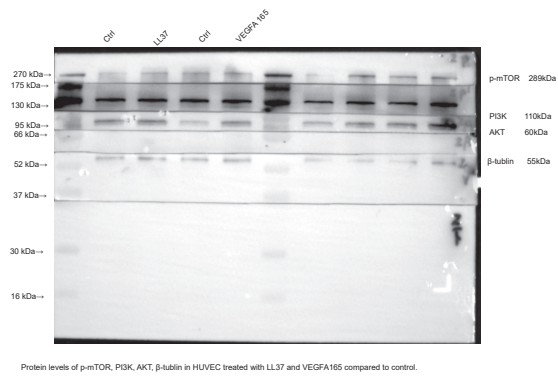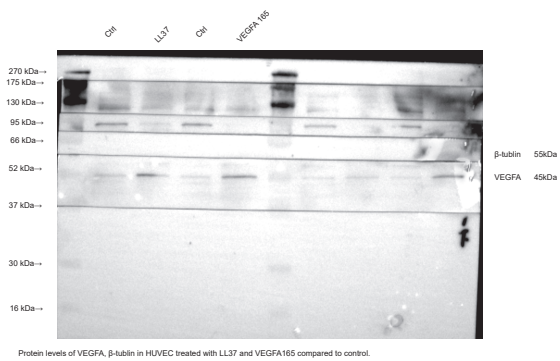

Supplement: Supplementary file 1 [file DataSheet2.pdf]
