# Supplementary material for: LL37 promotes angiogenesis: a potential therapeutic strategy for lower limb ischemic diseases
Source: Front Pharmacol. 2025 Apr 23;16:1587351. doi: 10.3389/fphar.2025.1587351 (PMC12055537; doi:10.3389/fphar.2025.1587351)
Supplement: Supplementary file 2 [file DataSheet1.pdf]

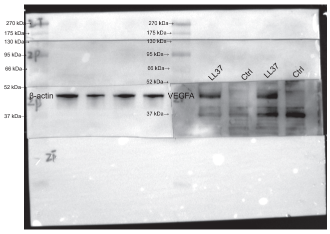

Protein levels of VEGFA in HUVEC treated with LL37 compared to control.

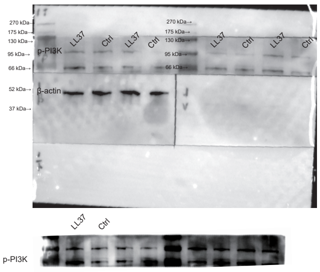

Protein levels of p-PI3K in HUVEC treated with LL37 compared to control.

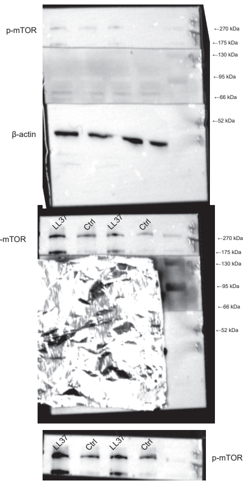

Protein levels of p-mTOR in HUVEC treated with LL37 compared to control.

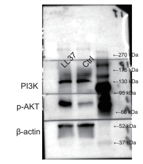

Protein levels of PI3K and p-AKT in HUVEC treated with LL37 compared to control.

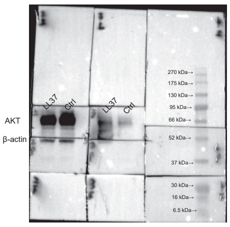

Protein levels of AKT in HUVEC treated with LL37 compared to control.

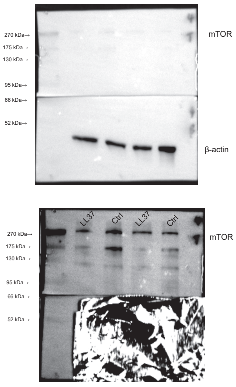

Protein levels of mTOR in HUVEC treated with LL37 compared to control.
